# Options to Improve the Action of PROTACs in Cancer: Development of Controlled Delivery Nanoparticles

**DOI:** 10.3389/fcell.2021.805336

**Published:** 2022-02-03

**Authors:** Alberto Juan, María del Mar Noblejas-López, María Arenas-Moreira, Carlos Alonso-Moreno, Alberto Ocaña

**Affiliations:** ^1^ Unidad NanoCRIB, Centro Regional de Investigaciones Biomédicas, Albacete, Spain; ^2^ Oncología Traslacional, Centro Regional de Investigaciones Biomédicas, Albacete, Spain; ^3^ Unidad de Investigación del Complejo Hospitalario Universitario de Albacete, Oncología Traslacional, Albacete, Spain; ^4^ Facultad de Farmacia de Albacete^,^ Universidad de Castilla-La Mancha, Albacete, Spain; ^5^ Experimental Therapeutics Unit, Hospital Clínico San Carlos, IdISSC and CIBERONC, Madrid, Spain

**Keywords:** PROTACs technology, drug delivery systems, nanomedicine, polymeric nanoparticles, lipid-based nanoparticles, metallic nanoparticles

## Abstract

Classical targeting in cancer focuses on the development of chemical structures able to bind to protein pockets with enzymatic activity. Some of these molecules are designed to bind the ATP side of the kinase domain avoiding protein activation and the subsequent oncogenic activity. A further improvement of these agents relies on the generation of non-allosteric inhibitors that once bound are able to limit the kinase function by producing a conformational change at the protein and, therefore, augmenting the antitumoural potency. Unfortunately, not all oncogenic proteins have enzymatic activity and cannot be chemically targeted with these types of molecular entities. Very recently, exploiting the protein degradation pathway through the ubiquitination and subsequent proteasomal degradation of key target proteins has gained momentum. With this approach, non-enzymatic proteins such as Transcription Factors can be degraded. In this regard, we provide an overview of current applications of the PROteolysis TArgeting Chimeras (PROTACs) compounds for the treatment of solid tumours and ways to overcome their limitations for clinical development. Among the different constraints for their development, improvements in bioavailability and safety, due to an optimized delivery, seem to be relevant. In this context, it is anticipated that those targeting pan-essential genes will have a narrow therapeutic index. In this article, we review the advantages and disadvantages of the potential use of drug delivery systems to improve the activity and safety of PROTACs.

## Introduction

**FIGURE 1:**
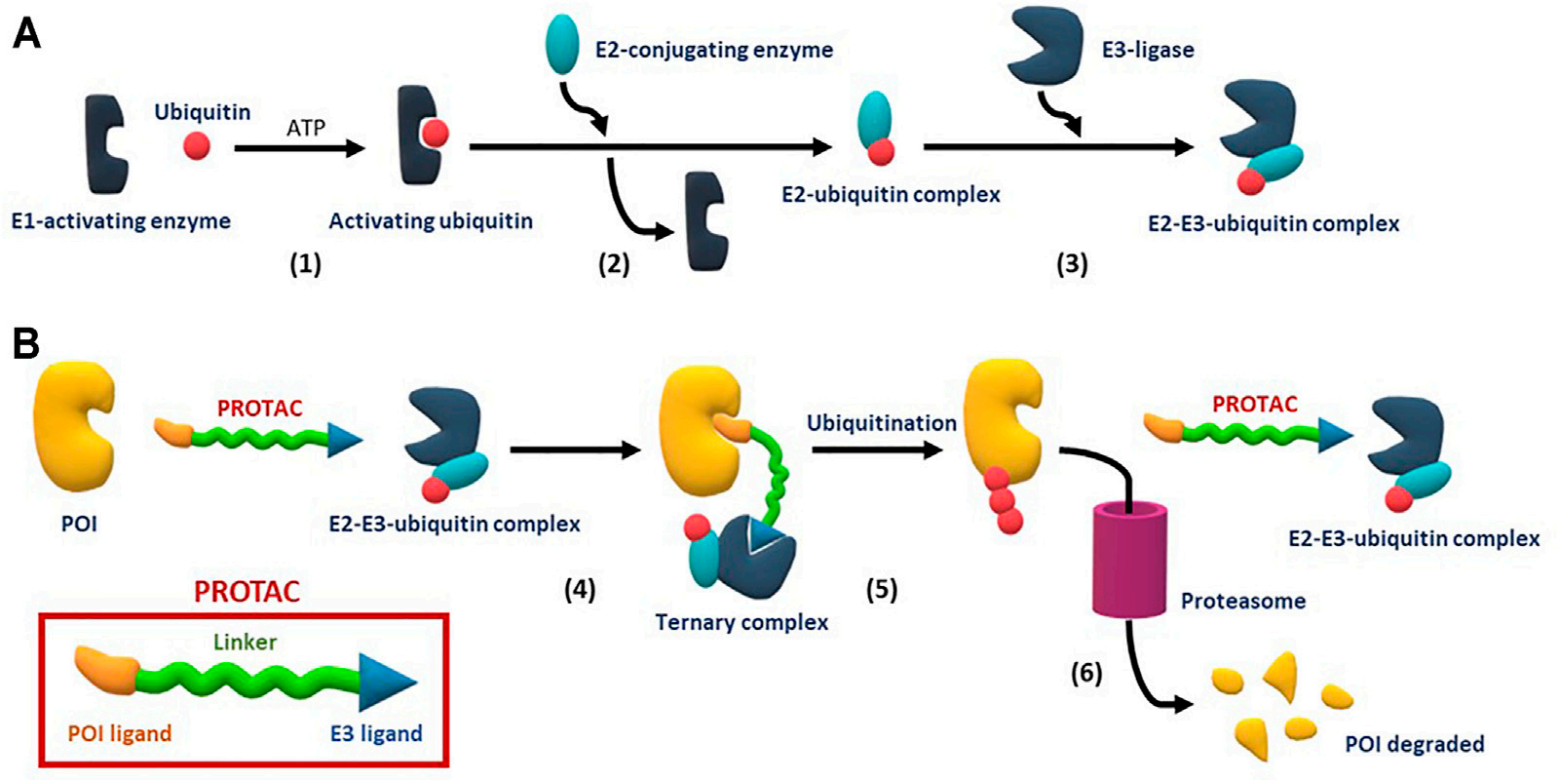
Representation of PROTACs structure (see inset red box), interactors, and mechanism of action. **(A)** Ubiquitination process: (1) Ubiquitin is activated by an E1-enzymatic protein in an ATP-dependent process, (2) the activated ubiquitin is transferred to a E2-conjugating enzyme, (3) the target protein is joined to the E2-ubiquitin *via* an E3-ligase enzyme, giving rise to an E2-E3-ubiquitin complex; **(B)** Mechanism of action of PROTACs divided into three steps: PROTACs are able to approach the protein of interest and the E3/E2/Ub complex forming ternary complexes (4), which allows the E3/E2/Ub complex to transfer ubiquitins to the target proteins (5) which, once tagged, are recognized by the proteasome for degradation (6).

PROteolysis TArgeting Chimeras (PROTACs) are bifunctional designed chemical structures that induce the degradation of target proteins. These compounds are composed of three elements, 1) a recognition molecule or ligand (warhead ligand) capable of binding specifically to a protein of interest (POI), 2) a chemical moiety that binds an E3 ubiquitin ligase (called ligase ligand), and 3) a linker that joins the recognition molecule and the E3-ligase (Figure 1) (Pandiella et al., 2015; Qi et al., 2021). The mechanism of PROTACs action was reported by Sakamoto et al. (2001), taking advantage of the ubiquitination-proteasome system (UPS) as described in Figures 1A,B (Zhang et al., 2021). The molecular basis of targeted protein degradation was recently discussed in detail by Dale et al. (2021).

**FIGURE 2:**
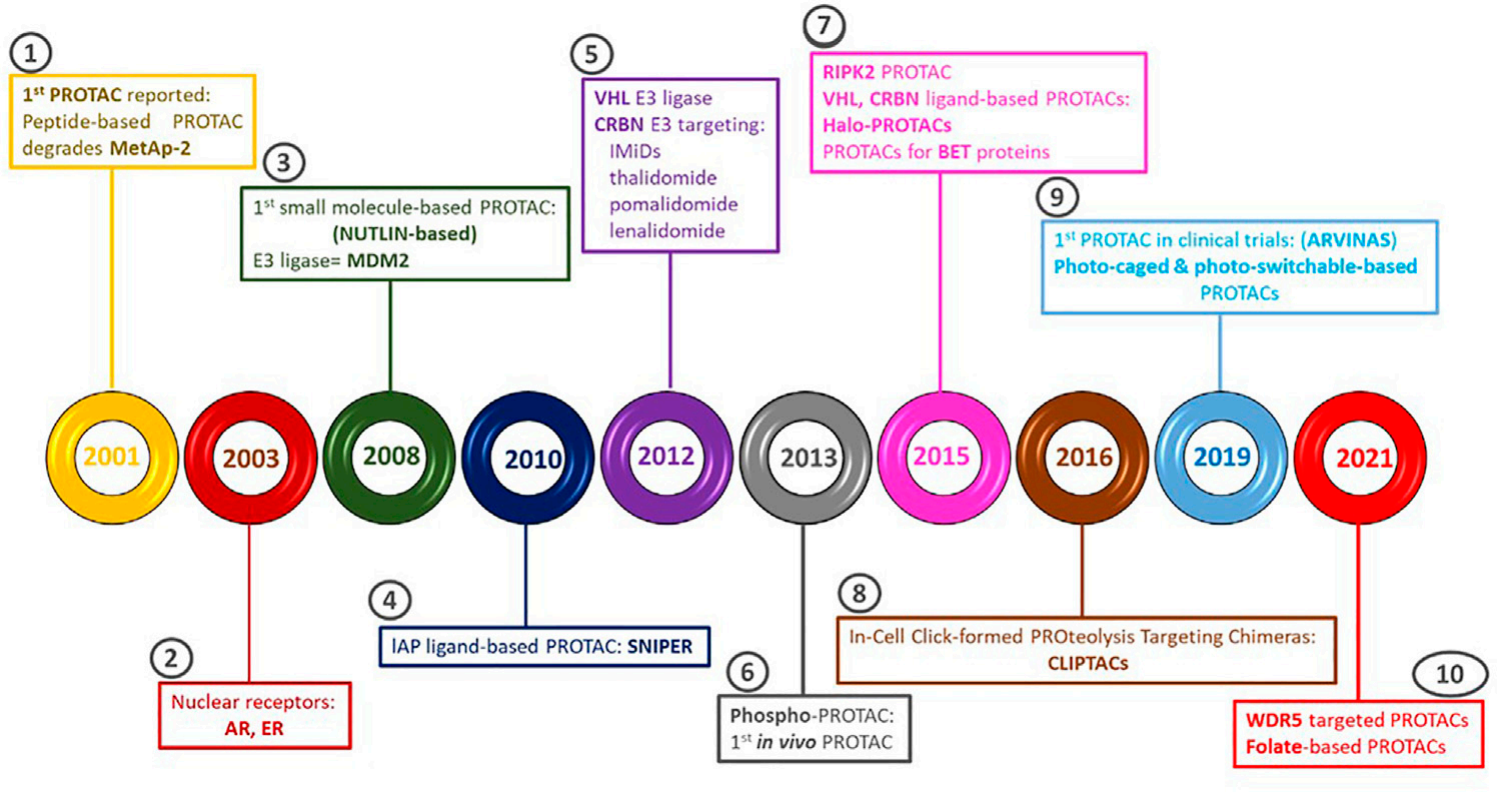
The 10 main events related to PROTACs technology development for clinical translation. (1)The first PROTACs technology based on a peptide was reported to degrade Methionine aminopeptidase 2 (MetaP2), an enzyme overexpressed in many forms of cancer (Sakamoto et al., 2001). (2) Novel PROTACs that induced degradation on androgen (AR) and estrogen (ER) receptors confirmed the proof of preclinical efficacy. (3) The first small-molecule PROTACs was reported in 2008, configured by murine double minute 2 (MDM2) as E3-ligase (Schneekloth et al., 2008). (4) Inhibitors of apoptosis proteins (IAP) were attained in the PROTACs structure as E3-ligase (Itoh et al., 2010). (5) Not only peptidomimetic ligands for VHL E3 were developed but CRBN E3 was identified as molecular targeting for immunomodulators, thalidomide, pomalidomide and lenalidomide (Ito et al., 2010; Itoh et al., 2010; Buckley et al., 2012). (6) The first PROTACs working *in vivo*, phosphor-PROTACs, were capable of inhibiting the tumoural growth in murine models, differentiating between various receptor tyrosine kinases (RKT) signalling routes (Hines et al., 2013). (7) The serine-threonine-protein kinase (RIPK2) receptor-interactor PROTACs that selectively induced degradation of RIPK2 at low doses were developed. Simultaneously, the usefulness of VHL E3 to facilitate guided degradation was demonstrated by its inclusion in HaloPROTACs. Additionally, PROTACs using Bromo and Extraterminal domains (BET) inhibitors targeting BET proteins, using both CRBN and VHL, were developed (Bondeson et al., 2015; Winter et al., 2015). (8) In-cell CLIck-formed Proteolysis Targeting Chimeras (CLIPTACs) were developed by Astex Pharmaceuticals (Lebraud et al., 2016). (9) Arvinas developed the first PROTAC tested in clinical trials. Photocontrol groups were incorporated into PROTACs (Xue et al., 2019) (10) New WDR5 targeting PROTACS were designed based on existing WDR5 ligands (Poso 2021), and folate based PROTACs were released to specifically deliver PROTACs in a controllable manner to degrade the POI, thus eliminating potential unwanted toxicity to normal tissues (Liu et al., 2021c).

Interest in PROTACs technology has grown in recent years. Zhou *et al.* in 2000 engineered the SCF E3 ubiquitin ligase complex to target pRb in yeast and human osteosarcoma SARS-2 cells (P. Zhou et al., 2000) and this achievement led to the development of PROTACs (Zou, Ma, and Wang 2019). Several groups including those leaded by Kathleen M. Sakamoto, Raymond J. Deshaires, Kyungbo Kim, Frank Mercurio, and Craig M. Crews dominantly contributed to the rapid development of PROTACs technology (Ocaña and Pandiella 2020). An overview of key achievements of this development is depicted in Figure 2. Updated lists of POIs, PROTACs, warheads, E3 ligands, and linkers are freely accessible at PROTAC-DB (http://cadd.zju.edu.cn/protacdb/) (Weng et al., 2021). The progress of PROTACs technology in the target protein degradation field and the biological effect of degradation is elegantly explained by Crews *et al.* (Alabi and Crews, 2021; Samarasinghe and Crews, 2021). An account of the most promising degraders in cancer are thoroughly discussed by Dale et al. (2021). Overviews of PROTACs to target cyclin-dependent kinase (CDK) (Rana et al., 2021), Kirsten rat sarcoma virus (KRAS) (Nagasaka et al., 2021), and Bruton’s tyrosine kinase (BTK) (Ran et al., 2021) were recently provided. Recent advances of VHL- and CRB-based PROTACs for treatment of diseases were summarized by C. Wang et al. (2021); 2022. The challenges of the emerging degradation platforms LYsosome-TArgeting Chimaeras (LYTACs) and Antibody-based PROTACs (AbTACs) to target extracellular and membrane proteins are described by Lin et al. (2021), and the advantages and disadvantages of light-controllable PROTACs for clinical application as a method of controlling induced protein degradation by light have been recently summarized by Liu et al. (2021a); Reynders and Trauner 2021.

Most of PROTACs currently under development are targeting cancer, neurologic disorders, and inflammation diseases. In relation to cancer, there is a great variety of PROTACs formulated against oncogenic proteins including transcription factors such as BET, STAT3, androgen, estrogen receptors; transmembrane receptors like, FLT-3, the epidermal growth factor receptor (EGFR); or intracellular signalling mediators, such as BRAF, KRAS, the fusion protein BCR-ABL, or the pro-survival protein MCL1, among others (Sun et al., 2019). The most common E3-ligases used for the generation of PROTACs are VHL and CRBN (Sun et al., 2019; Khan et al., 2020; Ocaña and Pandiella 2020). Of note, 17 of 18 PROTACs in clinical or preclinical development use CRBN and only one use VHL (Mullard 2021). On the other hand, the types of E3 binding used include prolines and amides, among others.

In 2019 two PROTACs (ARV-110 and ARV-471) patented by Arvinas entered the clinical phase. Phase 1 and 2 clinical trials to evaluate the safety, tolerability, and pharmacokinetics of ARV-471 alone (ARV-471 is administered once a day or twice a day for 28 days cycles) and in combination with Palbociclib (IBRANCE^®^) (daily oral doses of ARV-471 for 28 days in combination with palbociclib for 21 days) in patients with advanced or metastatic breast cancer, and ARV-110 (daily oral dosages once a day or twice a day in 28 days cycles) in patients with metastatic castration resistant prostate cancer are currently ongoing. ARV-110 targets the androgen receptor, a protein that contributes to the progression of prostate cancer (Neklesa et al., 2018), and ARV-471 targets the estrogen receptor, a transcription factor involved in the genesis and proliferation of most breast tumours. Both candidates can be taken orally and bind to the E3 ligase cereblon. Phase 3 studies in metastatic breast cancer are planned for ARV-471, including combinations with palbociclib, to be initiated in 2022.

## Advantages and Disadvantages of PROTACs Technology

PROTACs technology exhibits the potential to overcome the drawbacks of current cancer treatments by degrading targeted proteins. Chemotherapy, radiation therapy and surgery have been the traditional therapies for the treatment of cancer. However, chemotherapy also targets normal cells resulting in adverse side effects, surgery damages nearby tissues and radiation therapy can also cause damage to epithelial surfaces. To overcome these drawbacks, targeted therapy was proposed for selective elimination of cancer using small molecule inhibitors (SMIs) and monoclonal antibodies (mAbs). SMIs are low molecular weight molecules developed to inhibit enzymatic proteins such as those with kinase activity that have a specific catalytic site, with a potential oncogenic function. These agents have shown efficacy in a wide range of indications and clinical scenarios, particularly given the fact that whole sequencing studies are describing novel genomic alteration that translates into druggable oncogenic proteins (J. K. Smith, Mamoon, and Duhé 2003; Agafonov et al., 2015; Druker 2004; Esteban-Villarrubia et al., 2020). SMIs are smaller in size than mAbs and thus can easily permeate through plasma membranes. Furthermore, mAbs can only act on molecules expressed on the surface of cells or the extracellular matrix. SMIs are suitable for oral administration while mAbs are administered intravenously. However, SMIs have significant limitations: 1) lack of primary tumour responses due to primary resistant mechanisms (Ohashi et al., 2013), or the development of secondary resistance that led to a consequent relapse for patients with advanced cancer (Burrell and Charles, 2014). A huge proportion of these resistances is mediated by mutations at the kinase domain of the protein mainly for small kinase non-allosteric inhibitors (Rosenzweig 2012); 2) there are some “undruggable” proteins for SMIs, which comprises approximately 85% of all human proteins, including scaffold protein, transcription factor, cofactor, and other non-enzymatic proteins without catalytic site (T. K. Neklesa et al., 2017); 3) these drugs can produce on target off tumour toxicities acting of proteins involved in physiological functions. For instance, Anti-EGFR inhibitors can target the skin cells, which often express abundant EGFR, inhibiting normal growth (Widakowich et al., 2007). 4) Most SMIs have short bioavailability and require daily dosing.

Degradation of a target mutated protein could potentially overcome resistance in a very efficient manner, as has been demonstrated in preclinical models (Sun and Yu, 2020). In addition, early clinical evidence in trials using the androgen degrader has shown activity in those patients harbouring mutations at the androgen receptor that were resistant to androgen inhibitors. In line with this, PROTACs show activity with lower doses compared to the warhead ligand, as the occupancy of the target is not necessary to inhibit the activity and there is not a proportional dose-response relationship. However, for some compounds affinity of the chemical entity did not translate into more potency of the PROTACs as recruitment to the ligand PROTACs is also necessary. In addition, the antitumoural effect can last longer as there is a complete degradation of the target and therefore a longer time to recover, particularly for proteins with slow turnover. Bearing all of this in mind, PROTACs technology is currently under development with the aim to improve some of the limitations of classical SMIs (Table 1) (Burslem et al., 2018; Ocaña and Pandiella 2020).

**TABLE 1:** Advantages and disadvantages of PROTACs *versus* SMIs.

	Advantages	Disadvantages	
SMIs	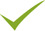 Good cell permeability	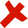 Cannot target ‘undruggable’ target	
	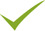 Solubility	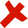 Drug resistance	
	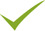 Oral administration	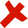 Adverse effects	
	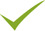 easily be combined with other treatments		
	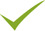 Broader range of clinical uses		
PROTACs	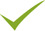 Can degrade ‘undruggable’ targets	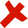 Insufficient understanding of the mechanism of action	
	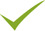 Overcome drug resistance	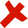 Design based on an empirical process	
	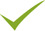 Requires low doses	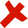 Unpredictive safety profile	
	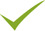 Oral administration	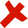 Poor cell permeability	
	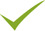 Longer effective period	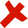 E3 ligase dependence	
	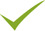 Better targeting ability	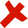 UPS dependence	

PROTACs technology requires a rational design. Although structural details of PROTAC-induced substrate recruitment to E3 ligases are reported, by solving ternary complex crystal structures or computational modelling, the molecular basis for target recruitment and formation of a ternary complex is not fully understood. Indeed, the formation of this ternary complex is key for the PROTAC to efficiently degrade the target protein. First, the design of PROTACs is an empirical process because not all ligases are compatible with all targets. Second, there is an insufficient understanding on the role of the linker in relation to the mechanism of action (Cyrus et al., 2011; Troup, Fallan, and Baud 2020). The linker length and composition are crucial parameters for a successful design of an effective PROTACs. A linker may provide the best chances of selectivity by minimizing degrees of freedom in the ternary complex while maintaining effectiveness (Bemis, Clair, and Burkart 2021). Other limitations of current PROTACS are based on both the selected POI and the targeted tumour type. Many of the PROTACs described at this moment target POIs considered as pan-essential genes (Ocaña and Pandiella 2020). These genes are mainly involved in relevant biological functions that maintain cell homeostasis and survival, including those present in cell cycle control, DNA repair or cell division, among others (Chang et al., 2021). These genes are widely expressed and, although upregulated in some tumours, their presence is significant and necessary for cells present in non-transformed tissues. Cells within high rate of proliferation including those at the epithelium harbor pan-essential genes. Examples include several CDKs, mitotic checkpoint proteins or transcription factors. Complete degradation of these proteins can induce severe toxicity and that could happen if a PROTAC target them, leading to a narrow therapeutic index. In this context, encapsulation of this type of PROTACs could undoubtedly improve efficacy and reduce toxicity. Novel formulations of this type of chemical entities have shown potential, and some of them like small molecules aurore kinase inhibitors are currently in clinical development (Floc’h et al., 2019). Another limitation of PROTACs is the ubiquitous expression of some of the POI in non-transformed tissues. Only targeting those proteins that are specifically expressed in tumoural tissue will augment the therapeutic index. As a proof of concept, compounds targeting the androgen and estrogen receptors have reached early-stage clinical development showing potential signs of activity and reducing toxicity as these two receptors are exclusively expressed in cancer cells. Other example of proteins specifically present in tumours are those that are mutated in some cancers like the SMARCA2 or SMARCA4 components of the BAF complex (Farnaby et al., 2019). Acting of proteins that are mutated or upregulated by gene amplification will potentially increase the therapeutic index, although this hypothesis has not been clinically tested. To optimize targeting, vectorization of PROTACs using antibodies against proteins exclusively expressed on the membrane of tumoural cells is an option that has shown to be effective in solid tumours with the use of antibody drug conjugates (ADCs) (García-Alonso, Ocaña, and Pandiella 2020; Manzano and Ocaña 2020). Similarly, one PROTAC ADCs has also been described (Maneiro et al., 2020).

Finally, PROTACs are large molecules without ideal molecular flexibility and water solubility which could hamper its oral absorption and cell permeability (Klein et al., 2020; Scott et al., 2020; Cecchini et al., 2021). Although most PROTACs in clinical trials are administered orally, their pharmacokinetic profile and metabolism could be further improved. For instance, novel oral formulations have been described (Wei et al., 2021), or improvements in parenteral administration could be achieved with the use of drug delivery systems.

## Nanomedicine to Overcome Current Limitations

Nanomedicine has raised many expectations to improve the treatment of cancer (van der Meel et al., 2019; Gonzalez-Valdivieso et al., 2021). It involves the use of nano-dimensional materials for diagnosis, and drug delivery (Ge et al., 2014; Chung, Leon, and Rinaldi 2019). In the field of drug delivery, the drug is encapsulated into nanoparticles (NPs) (Niza et al., 2021). The mechanism of action of such NPs is based on the enhanced permeability and retention effect (EPR) to favour its delivery to the site of interest by convection and diffusion processes (Yhee et al., 2013). The EPR effect is a heterogeneous phenomenon which is inter and intra tumoural dependent. Nevertheless, those NPs circulating long enough in the bloodstream will be internalized into the tumour cells by endocytosis, so endosomes coupled to lysosomes will cleave the NPs to release the free therapeutic agent into the cytoplasm. In this sense, there is still room to improve the controlled intracellular delivery of these compounds. For examples, some strategies are based on active targeting which rely on the ligand-receptor binding to improve accumulation of the nanodevices to targeted sites. On the other hand, other studies have indicated that escape from the endocytic pathway could improve the delivery of therapeutics (S. A. Smith et al., 2019). Nanomedicines already approved for clinical use were recently updated by Anselmo and Mitragotri (Anselmo and Mitragotri 2019). Table 2 depicted those nanomedicines on the market for the treatment of cancer.

**TABLE 2:** Type of anti-cancer nanomedicines on the market.

Drug delivery system	Therapeutic agent	Trade name	Clinical use
Lipid-based NPs	doxorubicin	Doxil^®^	ovarian cancer
		ThermoDox^®^	
		Myocet^®^	breast cancer
	daunorubicin and cytarabine	Vyxeos^®^	myeloid leukemia
	mifamurtide	Mepact^®^	osteosarcoma
	vincristina	Marqibo^®^	acute lymphoblastic leukemia
	cytarabine	Depocyt^®^	lymphomatous meningitis
	irinotecan	Onivyde^®^	pancreatic cancer
	paclitaxel	Lipusu^®^	lung cancer, breast cancer and ovarian cancer
Metallic Nps	radioenhancer	NBTXR3^®^	advanced sarcoma
Polymeric NPs	Aspargase	Oncaspar^®^	acute lymphoblastic leukemia
	leuprolide acetate	Eligand^®^	prostate cancer
Protein-based NPs	paclitaxel	Abraxane^®^	pancreatic cancer, breast cancer, non-small cell lung cancer
		Apealea^®^	ovarium cancer
	denileukin diftitox	Ontak^®^	T-cell lymphoma

In this sense, encapsulating PROTACs for the generation of “nanoPROTACs” would offer several advantages in comparison to conventional administration of PROTACs. Firstly, metabolism of PROTACs can be reduced, and lower doses would be required to be effective. In addition, metabolites will not be expected, avoiding unexpected activity or toxic side effects due to secondary compounds. Secondly, the controlled delivery of high concentrations of PROTACs by EPR effect or active targeting (guided NPs) might reduce their effects on non-transformed tissue. In fact, most NPs in clinical and preclinical development attenuate localization in healthy non-target tissues. Another advantage will be the improvement of cell permeability thanks to the mechanism of action of NPs based on the endocytosis and delivery by endosomal pathway.

Oral administration is the more convenient route of administration for cancer patients. Most PROTACs in preclinical and clinical trials are orally bioavailable. However, there are gastrointestinal biological barriers which decrease the bioavailability of PROTACs. In the last decade, nanomedicine has proposed various successful oral delivery systems to improve the bioavailability of orally administered therapeutics which could pave the way to address the clinical challenge for developing effective nanoPROTACs for cancer treatment (Parodi et al., 2021). Eudragit^®^ is a polymer coating designed for stabilizing oral formulations and proved to improve stability of liposomal formulations for oral drug delivery (Hua 2014). Functionalization of poly lactic-co-glycolic acid (PLGA) NPs allowed to specifically target intestinal transporters (Kou et al., 2017). The use of polycaprolactone for the generation of polymeric NPs increases the oral bioavailability of ellagic acid, a potent anti-cancer agent with very poor gastrointestinal absorption (Mady and Shaker 2017).

**FIGURE 3:**
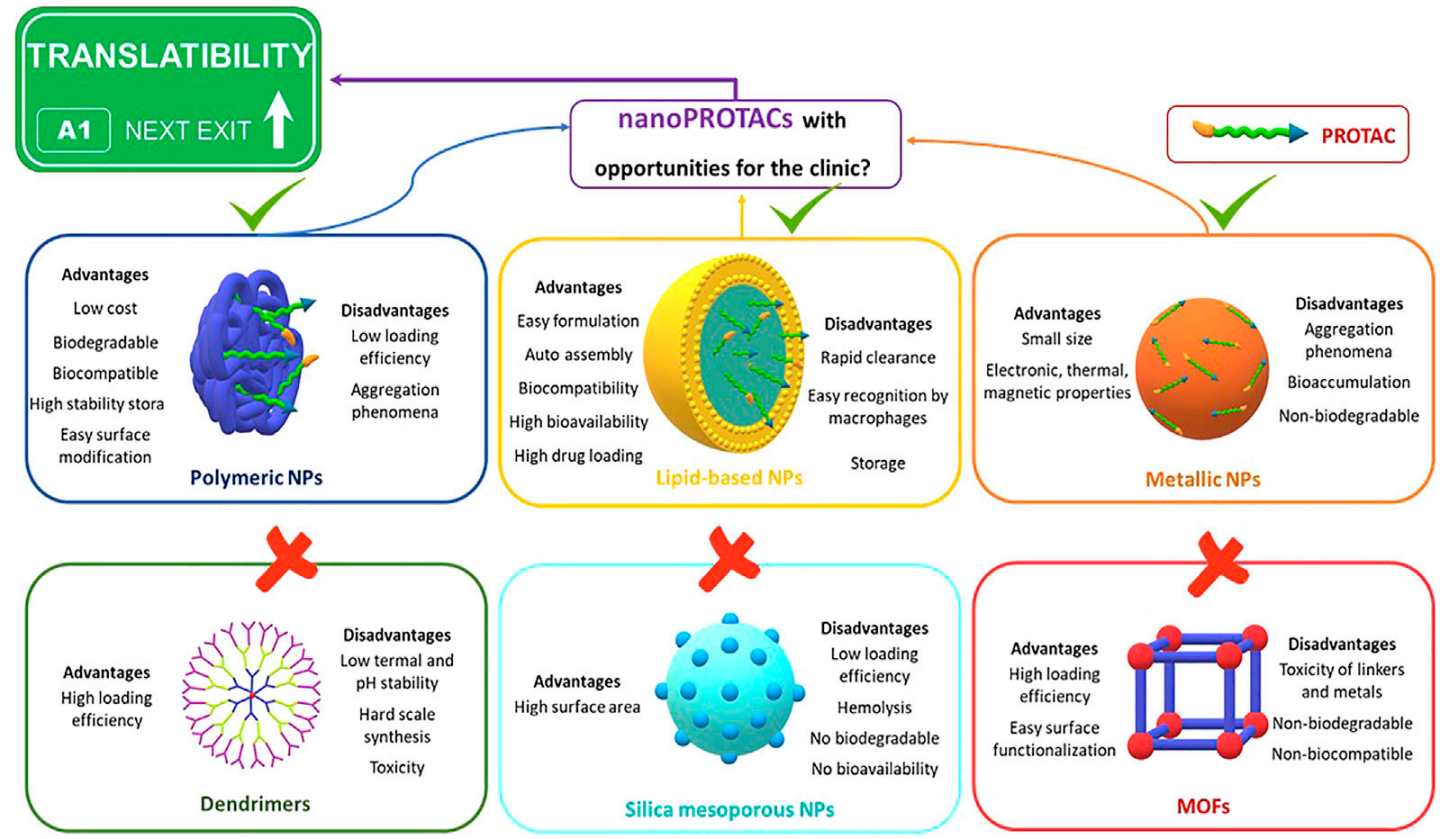
Advantages and disadvantages of different drug delivery systems for the rapid translatability of nanoPROTACs. MOFs and silica mesoporous NPs are not biodegradable and biocompatible, dendrimers are mainly toxic and difficult to scale their synthesis, metallic NPs are easily accumulated in the body and cause aggregation phenomena, lipid-based NPs reach high drug loading, but their half-life in blood might limit their clinical implementation, polymeric NPs are the most potential candidates for clinical translation due to their high biocompatibility, and payload and surface modification flexibility.

Currently, nanotechnology-based drug delivery systems can be formulated from soft (organic NPs) to hard materials (inorganic NPs). Nano-devices for drug delivery comprise a central material or matrix, a therapeutic payload and, in some cases, surface modifications. The development of a wide range of NPs capable of fitting the size, composition and functionality has provided an important resource for nanomedicine (Mitchell et al., 2020). With the aim of converting PROTACs into nanoPROTACs, some of the nanodevices might be circumvented for a rapid translation to the clinic (Mitchell et al., 2020) (Figure 3). First, dendrimers show a significant toxicity in many cases and their synthesis is difficult to scale (Janaszewska et al., 2019). Even though metal-organic frameworks (MOFs) and silica mesoporous NPs are flexible in terms of drug loading and surface modification, their non-biodegradability and toxicity are an impediment to a sustainable translation (Mitchell et al., 2020). Lipid-based, metallic and polymeric NPs are the most studied nanocarriers and, therefore, the most suitable candidates to generate nanoPROTACs.

## Lipid-Based NPs for the Controlled Release of PROTACs

Most common, lipid-based NPs are spherical platforms which contain at least a lipid bilayer that encompasses at least one internal aqueous compartment. As a delivery system, the lipid-based NPs have many advantages that include easy formulation, auto assembly, biocompatibility by parenteral administration, high bioavailability, capability of high payload transportation and a variety of controls to fit their biological, physical and chemical properties (Thi et al., 2021).

Lipid-based NPs are classified mainly into liposomes, transfersomes, niosomes, and solid lipid nanoparticles. Liposomes are aspherical vesicles composed of phospholipids and steroids, generally in the range of size from 50 to 450 nm (Tenchov et al., 2021). These are considered efficient vehicles for drug administration since their membrane structure is analogous to cellular membranes and facilitates cellular uptake (Thi et al., 2021). These nanostructures encapsulate hydrophilic and hydrophobic drugs and are biocompatible and biodegradable. Niosomes are spherical lipid-based NPs formulated using cholesterol and non-ionic surfactants (Bartelds et al., 2018). The stability of the nanocarriers is improved in comparison to liposomes but particle aggregation and drug leakage would hamper their use for PROTACs encapsulation (Bartelds et al., 2018). On the other hand, the use of phospholipids and edge activators lead to the formulation of transferosomes which would give better uptake (Rajan et al., 2011). However, these nanocarriers are very prone to oxidative degradation and their formulation implies a very high cost (Rajan et al., 2011). Finally, solid lipid NPs which need solid fats to be formulated show limitations to the clinic regarding drug loss within storage time (Montoto et al., 2020).

Concerning cancer therapies using lipid-based NPs, only liposomes have been approved by the Federal Drug Administration (FDA) (see Table 2). In this sense, Doxil^®^, a liposomal doxorubicin formulation functionalized with polyethylene glycol (PEG), was the first nanomedicine against cancer approved by FDA. Shortly afterwards, FDA approved other liposomal formulations such as liposomal daunorubicin (DaunoXome^®^), liposomal vincristine (Marqibo^®^) and, more recently, liposomal irinotecan (Onivyde^®^) (Mitchell et al., 2020).

Liposomes nanoPROTACs are the lipid-based NPs with more opportunities for a rapid clinical translation. Even though liposomes provide poor oral bioavailability, their low toxicity, affordable scale-up, and high loading efficiency set them as the most feasible option for a rapid translation to the clinic. Their low-cost formulation is very simple which is a benefit for the intended use. As well as for any other nanocarrier, they will be able to avoid unwanted PROTACs metabolization and improve biodistribution (Sercombe et al., 2015). However, liposomes’ half-life in blood is very short which implies a fast release of the PROTAC from the nanostructures (Sercombe et al., 2015; Guimarães, Cavaco-Paulo, and Nogueira 2021). Their stability *in vivo* is another significant limitation because it is affected by their size, superficial charge, lipidic composition, and number of layers. Of note, the incorporation into the nanostructure of a very high-volume molecule such as PROTACs will be a significant variable to consider for formulation. In this regard, surface modification (with ligands or polymers) to avoid an overly rapid absorption mediated by the reticuloendothelial system will be mandatory (Sercombe et al., 2015). Another aspect to consider is the administration route. PROTACs are orally administered and the efficient oral drug delivery through liposomes is a challenging task (Plaza-Oliver, Santander-Ortega, and Lozano 2021). Oral nanoPROTACs will have to deal with an acid environment and a significant enzymatic degradation to avoid solubilization of the carrier. Currently, there is a poor correlation between the *in vitro* and *in vivo* studies. Nevertheless, an important effort to optimize the encapsulation of PROTACs based on the ratio of solid/liquid lipids would be required (Montoto et al., 2020).

## Metallic NPs for the Controlled Release of PROTACs

Inorganic materials such as metals or silica have been used to synthesize nanostructured materials for various applications (Mitchell et al., 2020). Formulation of inorganic NPs must be accurate to obtain appropriate sizes, structures, and geometric shapes (Wu, Yang, and Wu 2016). Metallic NPs show different shapes: nanospheres, nanorods, nanostarts, nanoshells and nanoboxes. The matrix material itself gives them unique physical, electrical, magnetic, and optical properties. Most FDA-approved inorganic NPs are iron oxide NPs for iron replacement treatments. As representative examples, Ferinject^®^ is in the market as colloidal NPs consisting of a polynuclear iron (III)-(oxyhydroxide) core stabilized by carboxymaltose and proposed for the treatment of anemia (Wischmann et al., 2021), Feraheme^®^ are polymer coated supermagnetic iron oxide NPs formulated with mannitol indicated for the treatment of iron deficiency anemia in adult patients with chronic kidney disease.

Metallic NPs are widely explored for early detection, thermotherapy, and biomarker identification for the treatment of cancer. Regarding the manufacturing of drug delivery systems, gold and iron oxide are widely studied raw materials (Zhu et al., 2017; Dadfar et al., 2019). Even though gold NPs reported positive outcomes in preclinical studies as drug delivery systems for the delivery of the antitumour agent necrosis factoralpha, the FDA has not yet approved any gold NPs for cancer treatment. Hafnium oxide NPs (NBTX3^®^) is on the market indicated for head and neck cancer or non-small cell lung cancer (Gil et al., 2010; Rodriguez-Ruiz et al., 2019). Silver NPs, Ostim^®^, PerOssal^®^ and Vitoss^®^ are on the market as agents for radiotherapy. Magnetic iron oxide NPs composed of magnetite (Fe_3_O_4_) or maghemite (Fe_2_O_3_) display superparamagnetic properties to be successfully used as contrast agents, and stimuli-responsive drug delivery carriers. In this regard, NanoTherm^®^ therapy (Gil et al., 2010), based on injecting iron oxide NPs directly into the tumour and applying a magnetic field to selectively heat the NPs, is awaiting FDA approval. Nevertheless, the interest in metallic NPs is mainly focused on the development of diagnostic agents or hyperthermia treatment against tumours (Abbasi et al., 2021).

There are thus poorly reported efficient metallic drug delivery systems for cancer treatment (Abbasi et al., 2021). Formulation of metallic-based nanoPROTACs would give rise to drug delivery systems rapidly cleared by the reticuloendothelial system and, therefore, nanomedicines with little clinical value. In this regard, formulation of metal-based nanoPROTACs would require polymer conjugation to significantly decrease clearance rates. Overall, the high aggregation phenomenon disclosed for these nanocarriers along with the non-biodegradability and bioaccumulation may rule out these nanocarriers for a rapid translation of metal-based nanoPROTACs.

## Polymeric NPs for the Controlled Release of PROTACs

Polymeric NPs can be synthesized from natural material or synthetic polymers. Natural biodegradable polymers such as alginate or chitosan have been widely used to formulate polymeric NPs. These raw materials are biocompatible and biodegradable but face serious drawbacks for PROTACs encapsulation and their clinical translation (Gagliardi et al., 2021; Niza et al., 2021). The high immunogenicity of some natural polymers is a considerable limitation but the high variability in batch productions severely dampened the interest of researchers. Synthetic polyesters are biocompatible and biodegradable and can be designed to modulate loading, release kinetics, and stability (Niza et al., 2021). The most widely used polyester as raw material for NPs formulation is PLGA, due to its high biocompatibility and biodegradability. Indeed, it was approved by the FDA for clinical use (Lü et al., 2014). Polymeric NPs can be synthesized towards different techniques like emulsification, nanoprecipitation, ion gelation, and microfluidics, to produce NPs with different properties (Niza et al., 2021). The therapeutic agent can be physically retained or absorbed by the polymer matrix formulating nanospheres or can be dissolved entrapped in a shell disposed around an oily core to form nanocapsules. Both types of polymeric NPs allow the delivery of different payloads, including hydrophobic and hydrophilic compounds, as well as loading molecules with different molecular weights, like small molecules, biological macromolecules, proteins, and vaccines (Mitchell et al., 2020).

Currently, only a short quota of polymeric NPs has been approved by FDA and are clinically used for the treatment of cancer, but many polymeric NPs are being tested in large clinical assays. Of note, paclitaxel encapsulated in a polymeric micelle formulation, Genexol-PM^®^, is in clinical trials for several conditions, such as advanced urothelial cancer, metastatic and recurrent breast cancer and gynecological cancer (Kim et al., 2004; Lee et al., 2007; Ribas et al., 2010; Saif et al., 2010; Natale et al., 2014; Zuckerman et al., 2014; Autio et al., 2016; Jain et al., 2016; Von Hoff et al., 2016; Merle et al., 2017). PICN^®^, paclitaxel-loaded polymeric NPs of polylactide-polyethylene glycol, is being evaluated for metastatic breast cancer (Kim et al., 2004; Saif et al., 2010). Docetaxel was encapsulated in the same polymeric matrix, BIND-014^®^, and significant positive outcomes were reported for advanced metastatic cancer and non-small cell lung cancer (Kim et al., 2004; Lee et al., 2007; Jain et al., 2016). Doxorubicin was incorporated into a poly alkylcyanoacrylate matrix, livatag^®^, and tested for the treatment of hepatocarcinoma (Mura, Fattal, and Nicolas 2019).

Polymeric NPs seem to be the most promising drug carriers due to their nanoscale size and potential for the selective addressing and control delivery of PROTACs. These nanostructures are biodegradable, biocompatible, and highly stable during storage. The choice of the polymeric raw material for the generation of nanoPROTACs could provide flexibility in terms of physicochemical parameters, cargo, and release of PROTACs. The surface of polymer-based nanoPROTACs could be easily modified by using different polymer end groups or attaching other polymers in order to improve efficiency, making them useful for precision medicine (Mitchell et al., 2020). The route of administration of polymer-based nanoPROTACs would directly affect PROTACs bioavailability. Treatments based on polymeric NPs typically reported in preclinical studies and clinical trials require repeated doses administered by injection. Thus, parenteral route administration of nanoPROTACs would be expected for rapid translation to the clinic. However, oral administration would be desirable to guarantee patient endorsement (Liu et al., 2021b). Polymer-based nanoPROTACs formulation would be obtained in suspension to preserve aggregation phenomena after lyophilization. The suspension preparations should contain suitable antimicrobial preservatives, antioxidants or other excipients to aid stabilization. Polymeric NPs are stable in the gastrointestinal environment and would protect PROTACs from pH environments and enzyme degradation. Pegylation of nanoPROTACs will aim nanoPROTACs to cross the mucus barrier (Ensign et al., 2012). However, the particle size expected for polymeric-based nanoPROTACs will hamper effective transport across the intestinal epithelium. Several strategies would need to be pursued in order to overcome this limitation and make oral polymer-based nanoPROTACs feasible to the clinic (Pridgen, Alexis, and Farokhzad 2014). To date, oral administration of drug delivery systems is still a challenge and better understanding of the NPs transport through transmucosal barrier and epithelial absorption is required.

## Guided and Smart NPs for the Controlled Release of PROTACs

**FIGURE 4:**
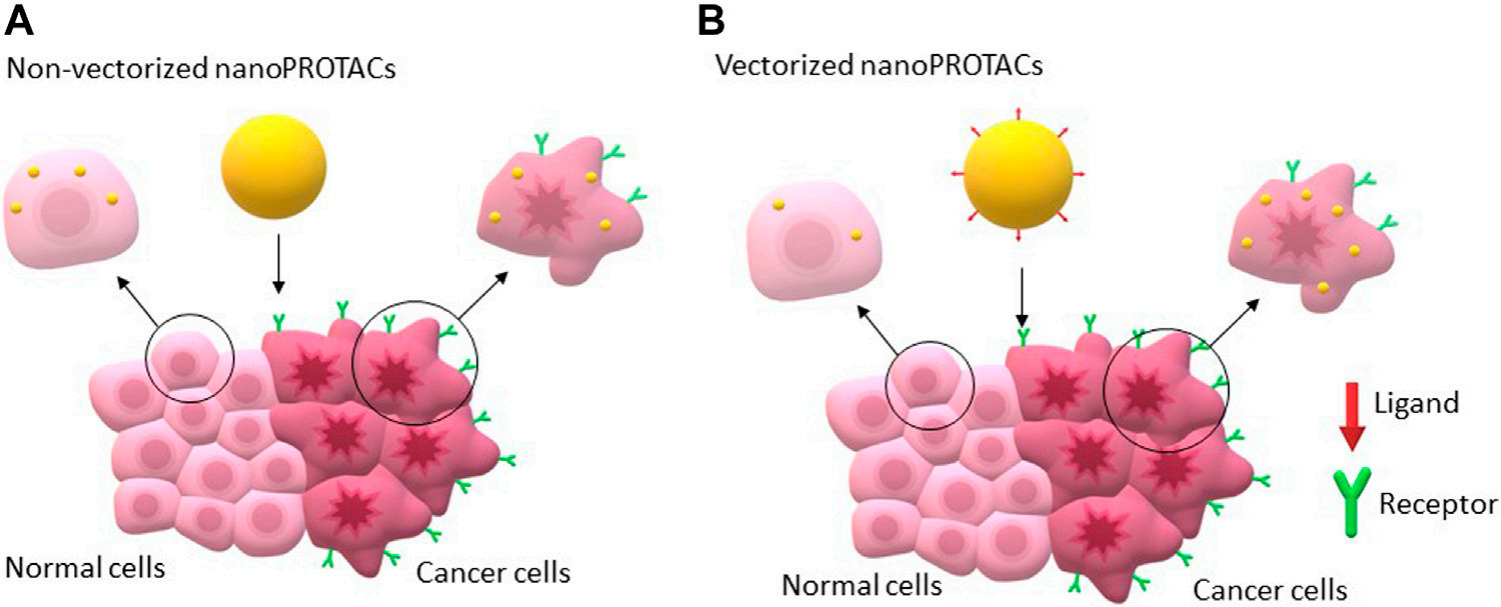
Illustration of how vectorized and non-vectorized nanoPROTACs would act on cancer cells.

As mentioned above, the design of PROTACs faces different limitations particularly when translating into clinical scenarios, where no target specificity exists, and a narrow therapeutic index could be anticipated, particularly if the warhead ligand targets pan-essential proteins. In this context, optimization of the delivery of PROTACs through the use of vectorized nanocarriers can augment efficacy preserving an adequate safety profile (Juan et al., 2020b; 2020a). Figure 4 illustrates the mechanism of action of vectorized NPs.

Different guided nanomedicines have been recently developed for the treatment of cancer (Juan et al., 2020b). In the field of inorganic nanocarriers, the characterization and *in vitro* evaluation of a nanoplatform based on Cer/Pom-PEG@GNPs, i. e, modified PEGs with ceritinib or pomalidomide on one side and a sulfhydryl group on the other, Cer- PEG-SH and Pom-PEG-SH, respectively, provided a potential new avenue for developing targeted ALK therapies (Y. Wang et al., 2020). The therapeutic ligands Cer- PEG-SH and Pom-PEG-SH were modified on the Gold NPs (GNPs) surface *via* a grating to methodology (A. M. Smith et al., 2015). GNPs were synthesized following the sodium citrate methodology (Frens 1973), Pom-PEG-SH was prepared by heating a mixture of 2,6-dioxopiperidin-3-yl-4-fluoroisoindoline-1,3-dione, H_2_N-PEG2000-SH and N,N-Diisopropylethylamine in N-Methyl pyrrolidone, and Cer-PEG-SH was prepared by stirring a solution of ceritinib triethylamine and SH-PEG2000-NHS in dichloromethane. Another example of smart inorganic nanocarriers has been recently applied in the development of a photodynamic therapy (PDT) approach based on ZnF16Pc-loaded and fibroblast-activation protein (FAP) specific single chain variable fragment (scFv)-conjugated apoferritin NPs (αFAP-Z@FRT) (S. Zhou et al., 2021). This approach has demonstrated an effective depletion of cancer-associated fibroblasts (CAFs) and an induction of an anti-cancer immunity. The combination of the αFAP PDT with αPD-1 antibodies leads to enhanced abscopal effects, suggesting the induction of an anti-CAF component in the cellular immunity by PDT treatment.

Lipid nanocarriers have been widely developed as immuno-carriers (Juan et al., 2020b; 2020a). Trastuzumab functionalized lipid-based NPs, loaded with antitumoural rapamycin and quantum dots as imaging agents were prepared for cancer therapy and imaging. Various *in vitro* studies were performed to evaluate their therapeutic efficacy over native drug and non-conjugated NPs in HER2+ SKBR3 breast cancer cell line (Parhi and Sahoo 2015). Recently, a study on bispecific antibodies lipid-based nanocarriers have highlighted the importance of simultaneous engagement of an immune cell and a cancer cell. This lipid-based phagocytosis nano-enhancer pointed the way for an improved tumoricidal efficacy by linking the immunological synapse and simultaneously engaging macrophages and cancer cells. Lipid-based NPs can increase anti-cancer efficacy by binding to SIPRα on macrophages and CD47 on cancer cells, at the same time as inhibiting CD47-SIRPα signalling pathway involved in the “do not eat me” signal (Ramesh et al., 2020).

In the field of polymeric NPs, guided nanomedicine systems have also been studied (Juan et al., 2020b; 2020a). A light responsive immunotherapeutic agent was developed for photothermal cancer immunotherapy. This nano-agent is based on a second near-infrared (NIR-II) photothermally activatable pro-nano agonist (APNA) triggering the release of covalently conjugated immunostimulants. Both the remote spatiotemporal control of immune activation and the immunogenicity mechanism during light irradiation and treatment were revealed and improved. APNA allowed for efficient tumour ablation and potentiated immunogenicity at up to 8 mm in deep solid tumours (Jiang et al., 2021). To investigate the anticancer activity of a BRD4 protein degrader (ARV), different nanoformulations were explored for parental delivery. PLGA-PEG NPs were produced to encapsulate lipophilic ARV for passive targeting and improving its cytotoxic effect (Van Hees et al., 2020). These NPs (ARV-NPs) were prepared by nanoprecipitation method by using a biodegradable PLGA-PEG polymer. Additional work demonstrated an ARV-loaded nanoformulation that was developed to improve the solubility, permeability, pharmacokinetics, and delivery of ARV had great translational potential for the treatment of drug-resistant and KRAS-mutant pancreatic cancers (Minko 2020).

The use of antibodies against membrane proteins expressed in tumoural cells as vectors *via* conjugation to NPs to deliver drugs in a controlled manner was reported by Ocaña *et al.* (Niza et al., 2019). This approach preserves the chemical structure, avoiding unpredicted metabolization and therefore reducing toxicity. The use of antibody conjugated NPs (ACNPs) might improve tumour accumulation, target selectivity, and cytotoxic efficiency for the treatment of breast cancer (Figure 4). Pioneering, PROTACs-loaded ACNPs conjugated with trastuzumab were developed, characterized, and evaluated *in vitro* against a panel of different breast cancer cells (Cimas et al., 2020). Polylactide NPs were prepared by nanoprecipitation and the displacement solvent method and trastuzumab was chemically conjugated to PEI coating NPs *via* carbodiimide chemistry. The PROTAC MZ1 was loaded into both NPs and ACNPs. Cytotoxicity studies indicated that MZ1-loaded ACNPs improved antitumoural effects in over-expressing HER2+ breast cancer cell lines in comparison to the non-vectorized nanoparticles and particularly to free MZ1 treatments. Remarkably, no additional toxicity of MZ1-loaded ACNPs was observed when compared to free MZ1.

## Future Strategies to Improve “nanoPROTACs”

PROTACs has several limitations that could potentially impact its clinical implementation. From the very first fact that they act on intracellular proteins limiting the targeting of extracellular proteins, to the circumstance that there is a small number of ligases for their potential optimization. In this context, although PROTACs against membrane receptors have been developed, for these compounds to work, the presence of intracellular UPS is needed, so proteins in the extracellular compartment cannot be targeted. In addition, targeted proteins are not only expressed in tumoural cells but in non-transformed tissue which could potentially lead to toxicity to non-tumoural tissue leading to a narrow therapeutic index.

It is important to mention that novel ways to exploit the protein degradation system are currently under early-stage investigation, including Lysosome targeting chimeras (LYTACs) or bi-specific targeting chimeras (AbTACs), that take advantage of the lysosomal pathway, or transmembrane E3 ligases, to act on membrane proteins. However, these approaches are beyond the scope of this review that aims to focus on how to improve the delivery of PROTACs. We suggest excellent reviews on this topic to the readers (Juan et al., 2020b; S.; Zhou et al., 2021; Ramesh et al., 2020; Jiang et al., 2021; Merle et al., 2017).

To augment the action of the compound on the tumour several strategies have been proposed. Our group has focused on the vectorization of PROTACs once loaded in NPs. This work has been described before in this review. This approach has potential benefits, as the specific guidance of the compound to tumoural cells avoids the action on cells not expressing the target. On the other hand, it displays some limitations, for instance, if there is a heterogeneous expression of tumour targets, a clonal expansion of cells non-expressing the target can limit the efficacy (Banik et al., 2020). In addition, antibodies due to their high molecular weight display limitations to penetrate in tumoural areas. These two problems could be overcome by binding two different antibodies to the nanoparticle against different targets expressed in tumoural cells, therefore reducing the expected resistance produced by the tumour heterogeneity. This bi-specific targeting has been developed with the use of bi-specific antibodies, some of them reaching the clinical setting (Ramesh et al., 2020). Regarding the high molecular weight, the use of a specific fraction of the antibody or the bind of a para-epitope (specific single chain variable fragment (scFv), to the particle could augment drug penetration as the molecular weight will be low. In this context, NPs loaded with PROTACs could be bound with two peptides designed against two different epitopes from a specific cell or group of cells. As this approach aims to be used as a carrier and a guided mechanism, no bell shape effect or valency optimization should be designed in advance. Improvements could be performed based on the different presence of targets. In addition, although it is anticipated a short pharmacokinetics profile, this will probably not be short enough to limit their clinical development. Finally, selection of a tumour specific E3 ligase has been reported as a mechanism to improve antitumoural activity reducing the potential side effects (Khan et al., 2019a). This specifically designed PROTACs with a selected E3 ligase could be incorporated in the bi-specific nanoparticle to improve efficacy.

Finally, for the successful translation of nanoPROTACs into the clinical setting, a strict non-clinical safety analysis, in addition to a chemical and manufacturing control procedure, should be developed and implemented (Germain et al., 2020). nanoPROTACs would require the assessment of their properties: particle size, polydispersity of formulation, surface charge, drug loading and efficiency, release profile and stability during storage, and safety and effective profiles: biodistribution, pharmacokinetics, metabolism, and immunological effects. Currently, only a few standard methods for the characterization of nanomedicines exist. In this context, there is a need to development a regulatory framework for nanomedicines (Bremer-Hoffmann, Halamoda-Kenzaoui, and Borgos 2018).

## Conclusion

In conclusion, the use of nanoparticles to improve the delivery and mechanism of action of PROTACs is a strategy that has potential to be further developed. First examples of how to optimize the protein degradation machinery using these agents has been described. However, optimization of chemical and manufacturing control procedures will be key for their future clinical development.
